# Efficacy of Polydextrose Supplementation on Colonic Transit Time, Bowel Movements, and Gastrointestinal Symptoms in Adults: A Double-Blind, Randomized, Placebo-Controlled Trial

**DOI:** 10.3390/nu11020439

**Published:** 2019-02-20

**Authors:** Alvin Ibarra, Tetyana Pelipyagina, Matthew Rueffer, Malkanthi Evans, Arthur C. Ouwehand

**Affiliations:** 1 DuPont Nutrition and Health, Danisco Sweeteners Oy. Sokeritehtaantie, Kantvik 02460, Finland; Arthur.Ouwehand@dupont.com; 2 KGK Synergize Inc. Suite 1440, One London Place, 255 Queens Avenue, London, ON N6A 5R8, Canada; tetyana@kgksynergize.com (T.P.); matthew@kgksynergize.com (M.R.); mevans@kgkscience.com (M.E.)

**Keywords:** polydextrose, transit time, bowel movements

## Abstract

The addition of fiber is one of the most important dietary means to relieve constipation through lifestyle modification. Polydextrose (PDX) has been reported in several studies to increase fecal bulk, soften stools, and increase the number of defecations. However, there are few studies on the effect of PDX on colonic transit time (CTT). Therefore, the aim of this study was to demonstrate the effect of PDX on CTT and other aspects of gastrointestinal function during two weeks (Day 1 to Day 14), preceded by a 2-week run-in period (Day -14 to Day -1). A total of 192 adults who were diagnosed with functional constipation per Rome III criteria were recruited for the study. Participants were randomized equally into 4 groups (12 g, 8 g, or 4 g of PDX or placebo per day). The primary endpoint was CTT, assessed using radio-opaque markers and abdominal X-rays on Day 0, the baseline; and Day 15, the end of the intervention. Secondary outcomes that were measured using inventories were the patient assessment of constipation symptoms and quality of life, bowel function index, relief of constipation, bowel movement frequency (BMF), stool consistency, degree of straining, and proportion of bowel movements. Ancillary parameters and harms were also evaluated. The recruited population was not sufficiently constipated (e.g., baseline values for CTT and BMF of 42 h and 8.7 BMF/week, respectively). Despite this limitation, our results demonstrated an increased number of bowel movements when supplemented with PDX at a dosage of 12 g per day for 2 weeks. This dosage also consistently improved the secondary outcomes that were measured using inventories at Day 15, compared with the baseline. No serious or significant adverse events were reported during the study.

## 1. Introduction

Constipation is a commonly diagnosed gastrointestinal disorder with an estimated prevalence in the general population of 12% to 19% [1,2]. The prevalence of constipation is likely to increase with the aging of the population in Western countries [3] and will otherwise be more prevalent with increased adoption of a Western lifestyle. Constipation results in a lower quality of life and significant health care costs to the individual and society [4]. Consumers in the USA and UK collectively spend nearly 1 billion US dollars annually for over-the-counter laxatives [5,6].

Chronic constipation is diagnosed almost solely based on patient-reported symptoms, which generally include unsatisfactory defecation due to infrequent stools, difficult stool passage, or both [7]. The cause of chronic constipation is multifactorial, with physiological changes, psychological factors, and lifestyle influences identified as possible contributing factors [7]. Consequently, the identification of effective constipation treatments remains a challenge. Evidence-based approaches to managing constipation include fiber, stimulant laxatives, polyethylene glycol, selective serotonin reuptake inhibitors, lubiprostone, and guanylate cyclase agonists.

The initial management of constipation is focused on evaluating lifestyle and diet variables as possible factors. The addition of fiber is one of the most important dietary means to relieve constipation [8]. Polydextrose (PDX) is a soluble food ingredient that cannot be digested by intestinal enzymes and thus potentially affects colonic function [9,10]. PDX has been reported in several studies to increase fecal bulk and soften stools [11,12,13,14,15,16,17,18,19]. Prebiotic fibers such as PDX also stimulate intestinal peristalsis and increase defecation frequency [13,14,20,21]. However, there are few studies on the effects of PDX on shortening total or colonic transit time (CTT), and the results have been inconclusive [13,18,19].

Historically, health care professionals have defined constipation as fewer than 3 bowel movements per week [22]. The Rome Foundation introduced a standard for classifying and diagnosing functional gastrointestinal disorders. The Rome criteria were developed for global adoption and use by physicians, pharmaceutical companies, and regulatory agencies [23,24]. The Rome III criteria encompass standards for diagnosing functional constipation, which extend to other symptoms besides reduced bowel movement frequency (BMF) [24]. In addition, there are standard outcome measures of constipation, such as objective measurements of CTT by radio-opaque marker intake and X-ray count [25] and the evaluation of constipation symptoms using validated questionnaires that are assessed by the subject or by health practitioners during medical visits [26,27,28,29].

The measurement of CTT is considered the standard method for examining bowel movements [20]. Notably, reported symptoms and quality of life ratings are not clearly or consistently related to a slow transit time but correlate consistently with an increase in fecal mass [18,19]. This raises a particular challenge for demonstrating shorter transit times by dietary means. Identification of a study population is usually based on symptom reporting, such as the so-called Rome criteria, but the preferred primary endpoint in a trial is based on standard methods and hard endpoints, such as transit time measurement, especially when substantiating a health claim.

Given the promising benefits of fiber supplementation on symptoms of functional constipation, the objective of this clinical trial was to evaluate the safety and effectiveness of supplementation with a proprietary PDX fiber product for 2 weeks, over a range of doses, on CTT and gastrointestinal symptoms in adults with functional constipation.

## 2. Materials and Methods 

### 2.1. Study Design

**Figure 1:**
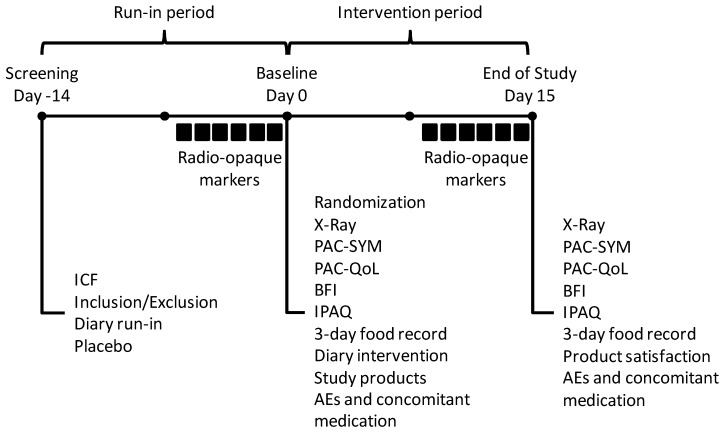
The study setup. AEs—adverse events; BFI—bowel function index; ICF—informed consent form; IPAQ—international physical activity questionnaire; PAC-SYM—patient assessment of constipation symptoms; PAC-QoL—patient assessment of constipation quality of life. ■, radio-opaque markers were consumed at the study site from Day -6 to Day -1 during the run-in period and from Day 9 to Day 14 during the intervention period.

This randomized, double-blind, placebo-controlled, parallel 2-week intervention study included 4 feeding groups over several doses (Figure 1). The study was conducted in full accordance with the Declaration of Helsinki and good clinical practice (GCP) standards [30,31] and was registered at ClinicalTrials.gov (NCT 02314936). All participants gave their written, informed consent and were told that they could withdraw from the study at any time. The study was performed in Ontario, Canada. The study protocol was reviewed by the Natural Health Products Directorate (NHPD), Health Canada, and a research ethics board. Unconditional approval was granted by the Institutional Review Board (IRB Services, Aurora, ON, Canada).

### 2.2. Study Subjects

A total of 192 participants aged 18–70 years were recruited from the area of London (ON, Canada) using a KGK Science in-house participant database (KGK Synergize Inc., London, ON, Canada), radio and newspaper advertisements, flyers, internet marketing, and website postings. They met the Rome III criteria for functional constipation (self-reported) in the last 3 months, with symptom onset occurring at least 6 months prior to the diagnosis [24] as follows: *a.* Two or more of the following criteria were met: *i.* straining during at least 25% of defecations; *ii.* lumpy or hard stools in at least 25% of defecations; *iii.* sensation of incomplete evacuation for at least 25% of defecations; *iv.* sensation of anorectal obstruction/blockage for at least 25% of defecations; *v.* manual maneuvers to facilitate at least 25% of defecations (e.g., digital evacuation, support of the pelvic floor); and *iv.* fewer than 3 defecations per week. *b.* Loose stools were rarely present without the use of laxatives. *c.* Insufficient criteria for irritable bowel syndrome—per Rome III criteria [24].

Other inclusion criteria were a body mass index (BMI) between 18.5 and 29.9 kg/m^2^, the ability to comprehend the full nature and purpose of the study, consent to the study and willingness to comply with study products and methods, and coverage by the health insurance system. In females of childbearing potential, a medically approved method of birth control and a negative urine pregnancy test were required.

We excluded subjects with one or more of the following criteria: Major gastrointestinal complications (e.g., Crohn’s disease, colitis, celiac disease); prior abdominal surgery that in the opinion of the investigator could have presented a risk for the subject or confounded study results; consumption of probiotics or prebiotics in the 2 weeks before screening or during the trial (other than study products); laxative use within 48 hours of screening; anticipated major dietary or other lifestyle changes during the study; systemic steroid use in the 1 month before screening; eating disorder; contraindication to dairy products (e.g., lactose); history of alcohol, drug, or medication abuse; pregnancy; planning pregnancy; lactation; participation in another study with any investigational product within 60 days of screening; belief of the investigator that the participant could be uncooperative or noncompliant and should therefore not participate in the study; subject under administrative or legal supervision; and subject who would have received, in Canadian dollars, the equivalent of more than 4500 euros as indemnities for participation in biomedical research within the 12 last months. 

In addition, the regular use of any drug or dietary supplement that affects intestinal transit (e.g., iron; opioids; sucralfate; misoprostol; 5-HT-antagonists; antacids with magnesium, calcium, or aluminum; antidiarrheal medication; anticholinergic agents; calcium or magnesium supplements; calcium channel blockers; tricyclic antidepressants; or NSAIDs) within 1 month before screening and during the trial was prohibited.

### 2.3. Study Products

Participants who passed the initial screening entered a 2-week run-in period (Day -14 to Day -1) and received 12 g maltodextrin sachets as placebo powder to be mixed daily with water. Participants were not informed about the nature of the product. After successfully completing the run-in period, at baseline (Day 0), eligible participants were randomly assigned to study product groups according to a computer-generated randomization list. Participants were provided sachets containing 12 g PDX powder (Litesse^®^ Ultra, Danisco USA Inc., Terre Haute, IN, USA), 8 g PDX and 4 g maltodextrin, 4 g PDX and 8 g maltodextrin, or placebo powder consisting of 12 g of maltodextrin, according to the group into which they were randomized. Participants were required to add the entire contents of their sachet to 250 mL water and consume the beverage daily for 2 weeks (Day 1 to Day 14) in the morning at breakfast.

Randomization was carried out by a computer system using block randomization lists and concealed allocation. The study products were labeled with an individual randomization number. The placebo product was matched to the PDX supplements and contained similar excipients to ensure that the study was double-blind. The identity of the specific product was blinded to participants, site staff, investigators, and the statistician. Investigators and study staff remained blinded to the study groups assignments until the database was locked and the data were analyzed. Randomized codes were stored in individual, sealed, opaque envelopes for each participant and were available to the principal investigator only in the case of severe adverse events (SAEs). Compliance was assessed by counting the number of returned empty sachets at the end of study (Day 15). Compliance was calculated by determining the number of sachets that were taken (empty sachets) over a given period, divided by the number of sachets that was expected to have been taken, multiplied by 100. In the event of a discrepancy between the information in the participant diary and the amount of study product that was returned, use was based on the product that was returned unless an explanation for how the product was lost was provided.

### 2.4. Study Outcomes

The primary endpoint of this clinical trial was CTT, which was assessed using abdominal X-rays on Day 0 and Day 15. Each participant ingested 24 radio-opaque markers (Sitzmarks^®^, Konzyl Pharmaceuticals Inc., Easton, MD, USA) each day for 6 consecutive days prior to abdominal X-rays. The markers were ingested at the same time each day, with a window of 1 hour prior to the ingestion time and 2 hours after the ingestion time being acceptable. If a participant did not ingest the marker within this window, it was counted as a missed dose. The numbers of markers in the right, left, and rectosigmoid colon were summed to yield a total marker count. Marker counts were identified by a single board-certified radiologist who remained blinded to participant group assignments. CTT was assessed using the following equation [32]:

*CTT* = *n_i_* × (*t*/*N*)

where *n_i_* is the number of markers observed by the X-ray, *t* is the time between the ingestion of markers in hours, and *N* is the total number of markers ingested each day. Thus, if markers are consumed at 24-h intervals and the number of markers per day is 24, CTT equals the total marker count on the X-ray. The secondary objectives of this clinical trial were measured using self-assessed validated questionnaires at baseline and at the end of study, or on a daily basis, if appropriate. The patient assessment of constipation symptoms (PAC-SYM), measuring the severity of constipation symptoms over the past 2 weeks, was completed on Days 0 and Day 15 [27]. The 12-question PAC-SYM survey consists of 3 subscales (stool symptoms, rectal symptoms, and abdominal symptoms) and has been demonstrated to be internally consistent, reproducible under stable conditions, valid, and responsive to change, thereby providing a comprehensive means of assessing the effectiveness of treatments for constipation [27].

Participant assessment of constipation quality of life (PAC-QoL) is a 28-question survey that measures the impact that constipation has had on daily life over the past 2 weeks [28]. The PAC-QoL questions comprise 4 subscales (worries and concerns, physical discomfort, psychosocial discomfort, and satisfaction) and an overall scale [28]. Participants completed the PAC-QoL on Day 0 and Day 15. Similarly, the bowel function index (BFI), a 3-item questionnaire that assesses ease of defecation, feeling of incomplete bowel evacuation, and personal judgment of constipation over the past 7 days were completed on Day 0 and Day 15 [29]. The total BFI score is the mean score of the 3 distinct components, graded on an analog scale (0 = easy/no difficulty/not at all, 100 = very strong/very difficult) [29]. Adequate relief of constipation, a single-question dichotomous (yes/no) tool that asks participants if they have experienced adequate relief of constipation symptoms over the past week, was completed on Day 0 and Day 15. Participants recorded the number of defecations per day (stool frequency), stool consistency using the Bristol stool scale (BSS) form [26], degree of straining (1, not at all; 2, a little bit; 3, a moderate amount; 4, a great deal; and 5, an extreme amount), sensation of complete bowel emptying (yes/no), and severity of abdominal discomfort and bloating severity (1, none; 2, mild; 3, moderate; 4, severe; and 5, very severe) in a daily diary during the 2-week run-in period and each day during the 2-week supplementation period. At the end of the supplementation period, participants were asked to rate their overall satisfaction with the study product’s ability to relieve their constipation symptoms on a 5-point ordinal scale.

Safety was evaluated by measuring whole blood hematology (hemoglobin, hematocrit, red blood cells, white blood cells, platelets, and c-reactive protein), serum variables (urea, creatinine, bilirubin, aspartate transaminase, alanine transaminase, gamma-glutamyltransferase, alkaline phosphatase, fasting glucose, and carbon dioxide; along with the minerals calcium, phosphate, potassium, sodium, and chloride), urine analysis (color, appearance, protein, glucose, ketone, blood, nitrite, bilirubin, leucocyte esterase, urobilinogen, specific gravity, and pH), and blood pressure and heart rate in the screening phase and at the end of the intervention period. In addition, weight and height were measured to calculate BMI. A 3-day food record was completed the week before baseline and the week before the end of study to monitor background diet before and during the intervention. Total calories, carbohydrate (g), fat (g), protein (g), fiber (g), and liquid intake (ml) were calculated. The International Physical Activity Questionnaire (IPAQ)-short version was self-administered on Day 0 and weekly thereafter through study completion on Day 15 [33]. Adverse events (AEs) and all other symptoms that were observed by the investigator or spontaneously reported by study subjects were systematically recorded.

### 2.5. Statistical Analyses

Calculation of the total sample size was based on statistical power analysis which was calculated assuming a decrease by 12.5 h in the primary outcome CTT for the mean transit time in each group versus the placebo. The power calculation was based on a previous study on constipation carried out by part of the study team [34]. With a probability of 80% at a significance level of alpha 0.05, we concluded that 192 randomized participants (48 per group) would be necessary, allowing for 10% subject attrition.

The primary outcome variable—changes in CTT from the baseline to the end of study versus the placebo—was compared between groups using analysis of covariance (ANCOVA). The dependent variable was the post-baseline value, the group was the factor of interest, and the value of the variable at the baseline visit was a covariate. One-way analysis of variance (ANOVA) was used to compare continuous endpoints across the 4 groups, followed by multiple comparison tests for statistically significant ANOVAs. ANCOVA models were used to examine the effects of the study product on continuous endpoints. The effects of categorical covariates on categorical endpoints were analyzed using the Cochran-Mantel-Haenszel test, and 95% confidence intervals (CIs) were calculated for each proportion. The secondary variables were reported using descriptive statistics and analyzed using similar methods as with the primary parameter. The safety analysis included all subjects who received investigational products, including the placebo.

The primary efficacy analysis population included all participants who consumed at least 1 dose of the study product, a randomized intention-to-treat (ITT) population. A secondary per-protocol (PP) efficacy analysis was also conducted (data not shown); this analysis population consisted of all randomized participants who consumed ≥ 80% of the assigned study product and 100% of the radio-opaque pellets in a timely manner.

Probability values *p* ≤ 0.05 are statistically significant.

## 3. Results

### 3.1. Participant Characteristics and Compliance

A total of 323 participants were screened; 192 participants, matched for age (average age 43 years) and BMI (25 kg/m^2^), were enrolled in the study (Table 1).

**Figure 2:**
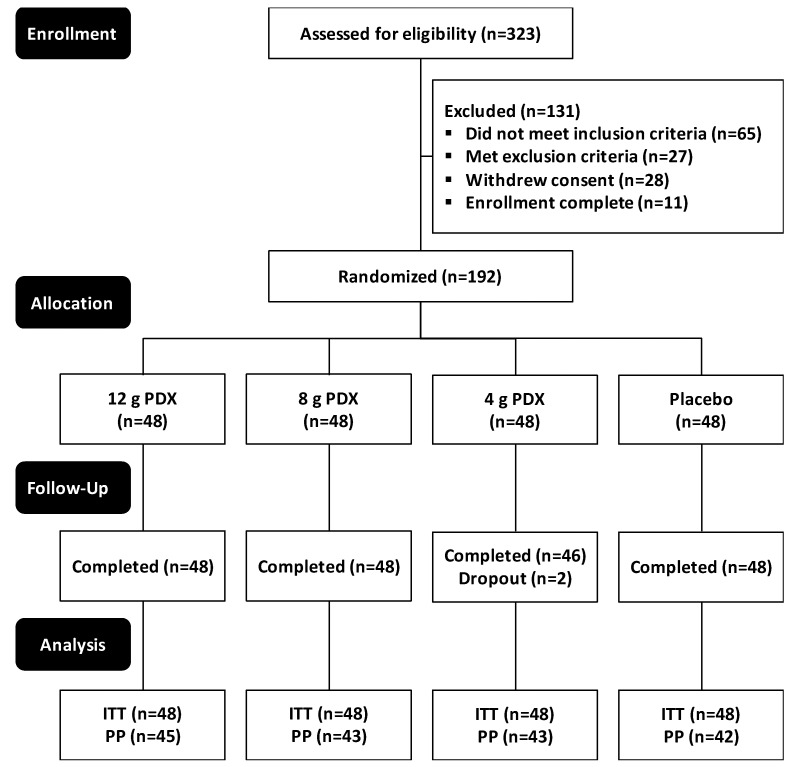
CONSORT flow diagram of the 2-week, 4-arm parallel-group (allocation ratio 1:1:1:1), double-blind, randomized, placebo-controlled, and monocenter study, preceded by a 2-week run-in period. CONSORT—consolidated standards of reporting trials; ITT—intention-to-treat population (all participants randomized at the second visit who consumed at least 1 dose of the study product); PP—per-protocol population (participants who attended the end-of-study visit and received at least 80% of the assigned study product and consumed 100% of radio-opaque markers during the intervention on time).

The eligible participants were randomized into 4 groups, with 48 participants in each group (Figure 2). The 4 groups received 12 g, 8 g, or 4 g of PDX or placebo. Two participants in the 4 g PDX group dropped out of the study between their baseline visit and end of study. Nineteen participants were excluded from the PP population. The results in the PP and ITT populations were similar; thus, we report the results from the ITT population as representative of all participants who enrolled in the study. Altogether, 190 participants completed the study, and all 192 participants were included in the ITT analysis. The intervention took place from 27 April 2015–15 April 2016. The study was finalized when the GCP study report was completed on 14 September 2016.

Participants were predominantly non-smoking, Caucasian females. All participants were deemed healthy per their laboratory results for safety parameters and blood counts. There were no statistical differences between the 4 study groups in demographics (age, BMI, alcohol use, smoking status, race, and ethnicity) at baseline. The product compliance was very good; the average compliance was >98% in all groups. The participants in this study had similar physical activity levels (data not shown) and food intake (Table 2) between groups.

The constipation status at screening was assessed for all participants through the self-reported Rome III criteria. All participants had two or more of the Rome III criteria, as required in the inclusion criteria to be deemed functionally constipated, and did not present with criteria that were sufficient for irritable bowel syndrome. Ninety-five percent of the participants reported the symptom of ‘lumpy or hard stools more than 25% of the time’ (*n* = 184), 94% reported ‘straining more than 25% of the time’ (*n* = 180), and 94% reported a ‘feeling of incompleteness more than 25% of the time’ (*n* = 181). At the baseline, an average value for CTT was 42 hours for all participants, and there were no statistical differences between the 4 study groups.

The average values for baseline PAC-SYM and PAC-QoL were 1.07 ± 0.56 and 1.10 ± 0.43, respectively. The average total score for the BFI of all participants at baseline was 44.9 ± 25.6. At baseline, 49.7% of participants experienced relief from constipation in the previous week, per the adequate relief questionnaire. The average reported BSS was 3.29, thus indicating harder-than-optimal stools. On average, participants presented at baseline with slightly more than 1 bowel movement per day. There were no statistical differences between the 4 study groups in baseline characteristics.

### 3.2. Colonic Transit Time (CTT)

There were no significant differences between groups in total CTT in the study populations at the end of the intervention period (Table 3). For total CTT, there were also no significant within-group changes from baseline. 

### 3.3. Patient Assessment of Constipation Symptoms (PAC-SYM)

Results of PAC-SYM are presented in Table 4. The PAC-SYM rectal symptom was the only subscale that showed between group differences. On Day 15, the 4 g PDX per day participants reported a significant 65% increase (*p* = 0.034) in their average PAC-SYM rectal symptom subscale compared with the placebo group (Dunnett-adjusted *p* = 0.027). Within groups, participants supplemented with 12 g PDX reported significant decreases in their PAC-SYM total (*p* = 0.004), rectal symptom subscale (*p* = 0.035), and stool symptom subscale scores (*p* = 0.0012) from the baseline to Day 15. Participants in the 8 g PDX per day group reported significantly decreased end of study measurements in PAC-SYM total (*p* = 0.003), abdominal symptom subscale (*p* = 0.014), rectal symptom subscale (*p* = 0.011), and stool symptom subscale scores (*p* = 0.008) versus baseline. Participants who consumed PDX at a rate of 4 g per day reported significant decreases in their stool symptom subscale (*p* = 0.029) from the baseline to Day 15. Finally, participants in the placebo group reported significantly decreased PAC-SYM total (*p* = 0.004), abdominal (*p* = 0.016), rectal (*p* = 0.017), and stool symptom subscales (*p* = 0.007) from baseline to the end of study.

### 3.4. Patient Assessment of Constipation Quality of Life (PAC-QoL)

Results of PAC-QoL are presented in Table 4. There was a significant difference between groups in the satisfaction subscale on Day 15 (*p* = 0.006); however, none of the PDX groups differed significantly from the placebo group in pairwise comparisons (all Dunnett-adjusted *p* = 0.062), despite the satisfaction subscale improving significantly in the groups with the highest doses of PDX (8 and 12 g) as compared to the baseline. Within groups, the PDX 12 g group reported significant decreases from the baseline in the PAC-QoL worries and concerns subscales (*p* = 0.005) and the physical discomfort subscale (*p* = 0.008), in addition to demonstrating a significant rise in the satisfaction subscale after 2 weeks of supplementation (*p* = 0.010). The 8 g PDX group reported significant declines from baseline measurements in worries and concerns (*p* = 0.004) and physical discomfort subscales (*p* = 0.019) and significantly higher satisfaction subscale scores (*p* = 0.004) after 2 weeks of supplementation. The 4 g PDX group significantly decreased from baseline to Day 15 on the worries and concerns scale (*p* = 0.046). The placebo group fell significantly from the baseline to the end of study in PAC-QoL overall score (*p* = 0.039) and the physical discomfort subscale (*p* = 0.047).

### 3.5. Bowel Function Index (BFI)

There were no significant between-group differences in the BFI questionnaire. Results are presented in Table 5 for the total score. Within groups, the 12 g PDX group experienced significant decreases from baseline to the end of study in total score (*p* = 0.028), feeling of incomplete bowel evacuation (*p* = 0.041), and judgment of constipation (*p* = 0.036). The 8 g PDX group underwent a significant decline from baseline to Day 15 in total score (*p* = 0.024). In the 4 g PDX group, total score (*p* = 0.005), and the feeling of incomplete bowel evacuation (*p* < 0.001) fell from the baseline to the end of the intervention. The placebo group reported significant decreases from the baseline to the end of study in total score (*p* =0.001), ease of defecation (*p* = 0.005), and judgment of constipation (*p* = 0.001).

### 3.6. Constipation Relief Questionnaire

There was a significant difference in the proportion of participants who experienced relief from constipation on Day 15 (*p* = 0.020); however, none of the participants who consumed the PDX product differed significantly from the placebo group. Within groups, the 12 g PDX (*p* = 0.009) and placebo groups (*p* = 0.031) had a significant increase in the proportion of participants who experienced relief from constipation relative to the baseline (Table 6).

### 3.7. Bowel Habits Diary

**Figure 3:**
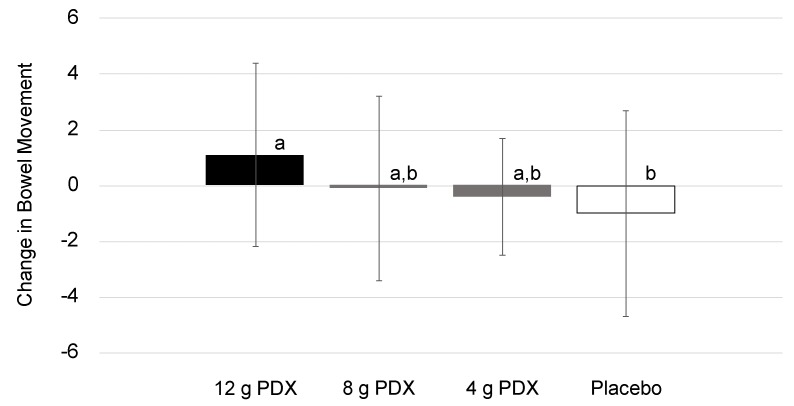
Changes in bowel movement from the baseline to the end of study (*p* = 0.017). Bars with letters are significantly different after Dunnett’s correction (*p* ≤ 0.05).

Results on bowel habits are presented in Table 7. During the 2-week supplementation period, the participants who consumed 12 g PDX per day recorded a significantly higher number of bowel movements (*p* = 0.017) per week compared to the placebo (Dunnett-adjusted *p* = 0.006) (Figure 3). Within groups, participants in the 12 g PDX group had a significant decrease from the baseline in the degree of straining (*p* = 0.002) and a significant rise in the proportion of complete bowel movements (*p* = 0.027) by the end of the intervention. The 8 g PDX group experienced a significant decline from the baseline in the degree of straining (*p* < 0.001) and a significant increase in the proportion of complete bowel movements (*p* = 0.007) by the end of the intervention. In the 4 g PDX group, stool consistency improved significantly, as measured with the BSS, at week 1 relative to the baseline (*p* = 0.019); yet, the average BSS did not change from 3. Participants in the placebo group had a significant decrease in the number of bowel movements during week 2 versus the baseline (*p* = 0.031).

### 3.8. The Severity of Abdominal Discomfort and Bloating

There were no significant between-group differences in the abdominal discomfort or bloating questionnaire. The 8 g PDX group demonstrated a significant within-group decrease from the baseline to the end of the intervention for the severity of bloating (*p* = 0.003) (data not shown).

### 3.9. Product Satisfaction

There was a significant between-group difference in product satisfaction on Day 15 (*p* = 0.012), wherein participants who consumed 12 g PDX per day reported significantly greater product satisfaction than the placebo group (Dunnett-adjusted *p* = 0.040) (data not shown).

### 3.10. Safety Parameters

Concerning laboratory safety measures, several differences were found between groups, but all hematology and clinical chemistry changes were considered clinically insignificant and were within the reference values. There were no significant differences between groups in urinalysis, heart rate, or blood pressure. Within groups, all groups experienced a significant increase in systolic blood pressure from the screening. The 12 g PDX group also had a significant increase in diastolic blood pressure (*p* = 0.015), and the placebo group had a significantly higher heart rate (*p* = 0.043) relative to the baseline. Weight and BMI remained similar throughout the study for all groups (data not shown).

Ninety-three AEs were recorded by 65 participants over the duration of this study. Of these AEs, 30 were in the 12 g PDX per day group, 23 were in the 8 g PDX per day group, 11 occurred in the 4 PDX g per day group, and 29 developed in the placebo group. Of the 93 reported AEs, 47 were assessed as being unlikely related, and 18 were considered to be unrelated to the investigational product. The 18 AEs that were considered likely to be related to PDX were abdominal discomfort (2), abdominal distension (3), abdominal pain upper (3), constipation (1), flatulence (2), frequent bowel movements (1), nausea (2), pruritus (1), diarrhea (1), dyspepsia (1), and vomiting (1). Of these AEs, 9 were mild, 8 were moderate, and 1 was severe (nausea). The 10 AEs that were assessed as probably related to the placebo were abdominal discomfort (2), abdominal distension (2), abdominal pain upper (3), influenza-like illness (1), and nausea (2). Of these AEs, 3 were mild and 7 were moderate, with 1 AE requiring concomitant medication. All AEs were resolved by the end of study. No SAEs or significant AEs were reported during the study.

## 4. Discussion

The initial management of constipation symptoms is focused on evaluating lifestyle and diet variables as possible culprits [35]. If lifestyle modifications are unsuccessful in alleviating constipation, various medications may be prescribed [35]. Overall, these medications have limited efficacy, are expensive, and may result in adverse side effects, especially over the long term [35]. Approximately 50% of adults with constipation are not completely satisfied with available treatments [35]. Consequently, there is a clear need for alternative constipation treatments that are safe, effective, and cost-effective.

We conducted a clinical trial to assess the effects of 4, 8, and 12 g PDX per day in participants who were diagnosed with functional constipation per Rome III criteria [24]. We have earlier reported that using Rome III as the sole criteria to diagnose functional constipation may produce several subgroups, creating a lack of homogeneity that could have significant disadvantages in clinical research [36]. Accordingly, we observed important deviations from a typical constipated population at the baseline. For instance, using Rome III resulted in a baseline CTT of 42 h, whereas a meta-analysis reported 58 h (95% CI: 50–65 h) for a typical constipated population [37]. Similarly, other indicators of constipation at baseline were not typical, such as overall PAC-SYM and PAC-QoL scores of 1.07 and 1.10, respectively, whereas meta-analyses have reported higher values of 1.70 (95% CI: 1.58–1.83) and 1.97 (95% CI: 1.70–2.24), respectively, for constipated populations [37]. Certainly, one of the most relevant parameters for defining constipation is stool frequency, which in our study was 8.7 bowel movements per week, an extremely high value compared with 2.7 bowel movements per week in a meta-analysis (95% CI: 2.4–3.0) [38]. This strongly suggests that the population in our study was not sufficiently constipated, which is likely to limit our interpretation of the effects of PDX on constipation. Otherwise, the population in this study was homogeneously distributed with regards to demographics and dietary intake.

Although the primary endpoint of CTT did not differ significantly between groups, it is important to note that changes of any size or direction may play an important clinical role in functional constipation. Participants who consumed 12 g PDX per day experienced a 2-h reduction in CTT, whereas those who consumed 8 or 4 g PDX and the placebo group experienced increases from 2–4 h. A previous study reported the opposite effect during 4-week administration of 12 g PDX per day on a constipated population, wherein PDX increased CTT, whereas the placebo reduced it [39]. However, baseline CTT values were non-homogeneous in that study, with an average CTT of 64 h for the placebo and 51 h for the 12 g PDX group; thus, the placebo had more room for improvement than the PDX group, requiring cautious interpretation of these results [39].

Although the PAC-SYM and PAC-QoL values were not sufficiently elevated at the baseline, PDX had positive effects on several scores. The PAC-SYM total score improved significantly from Day 0 to Day 15 in the groups that consumed 12 and 8 g PDX per day, although the placebo also improved this parameter. Similar effects were seen for the PAC-SYM abdominal symptoms score and rectal symptoms score for these 2 groups. All groups, including the placebo group, had an improved PAC-SYM stool symptoms score on Day 15 compared with the baseline value. Similar results were observed for the PAC-QoL scores, wherein all PDX doses and placebo improved the worries and concerns score versus the baseline. A comparable effect was observed for the PAC-QoL physical discomfort score, which improved in all groups, except the 4 g PDX per day group, compared with baseline. The groups that consumed 12 and 8 g PDX per day were the only groups to improve their PAC-QoL satisfaction scores among participants who reported functional constipation.

The overall improvement in symptoms of constipation was also reflected in the BFI scores for all groups, including the placebo. Although there appears to be a dose-response effect of PDX on weekly bowel movement frequency, this did not reach statistical significance; which may correlate to the low incidence of constipation in the study population. This tendency was also captured in the number of participants who declared a higher relief of constipation score, with 79% of participants who consumed 12 g PDX reporting a positive outcome after 2 weeks of intervention.

Despite the high stool frequency in the entire population at the baseline, the group that consumed 12 g PDX per day significantly increased their stool frequency by more than 2 bowel movements per week compared with placebo. These improvements were accompanied by a greater reduction in the degree of straining and a higher proportion of complete bowel movements after 2 weeks of consuming 8 or 12 g PDX. According to the US Food and Drug Administration, a commonly accepted, clinically relevant improvement in stool frequency for studies on functional constipation is ≥1 complete spontaneous bowel movements (CSBM) per week [40]. Similarly, the European Medicines Agency (EMA) guidelines recommend that the primary outcome of analysis be based on “responder analysis”—i.e., a comparison of the percentage of “responders” between groups [41]. It is important to highlight that according to the EMA, “responders” are defined as individuals who experienced at least 3 CSBMs/week and an increase of at least 1 CSBM/week compared with the baseline period for at least 75% of the duration of the study and for the last 4 weeks of intervention [41]; in comparison, the baseline for bowel movements in our current study was elevated. 

Nevertheless, increasing stool frequency without causing diarrhea in a general population is considered to be physiologically relevant for applying European Food Safety Authority health claims, within the scope of maintaining normal defecation [42]. Moreover, the use of laxatives and fiber can lead to an overall increase of 1.4 bowel movements per week, which may be considered to be clinically meaningful [22]—a threshold that was exceeded in our study by intervention with 12 g PDX per day. Subjects suffering from constipation are seeking relief in the short term [43]. Furthermore, earlier studies have shown that fiber, and in particular PDX, is able to influence bowel function within 2 weeks [13]. In this regard, PDX may offer relief of constipation in a reasonable period.

In our study, we observed an increase in weekly bowel movements with 12g PDX, but no diarrhea was reported as an AE, while other AEs did not differ in occurrence as compared to the placebo group, demonstrating that this dose of PDX is safe in adults. These findings are consistent with previous reports indicating that dietary fibers help to normalize bowel function [44] both in constipation and diarrhea.

## 5. Conclusions

In conclusion, despite the lack of a properly constipated population at the baseline (e.g., baseline values for CTT and BMF of 42 h and 8.7 BMF/week, respectively), our results demonstrated more bowel movements on supplementation with PDX at a dosage of 12 g per day for 2 weeks. Based on these results, future studies on PDX should investigate dosages at or near 12 g per day in a homogeneous constipated population. Further, extending the length of the intervention to increase the possibility of measuring efficacy outside of the placebo effect window is certainly warranted.

## Figures and Tables

**Table 1:** Demographics for participants in the intention-to-treat population (*n* = 192).

	All (*n* = 192)	12 g PDX (*n* = 48)	8 g PDX (*n* = 48)	4 g PDX (*n* = 48)	Placebo (*n* = 48)	*p*-Value σ
Age Mean ± SD	42.7 ± 18.8	42.9 ± 16.3	4297 ± 12.5	41.5 ± 17.1	43.6 ± 13.4	0.912 §
BMI (kg/m^2^) Mean ± SD	25.28 ± 2.85	25.27 ± 3.06	25.31 ± 3.09	25.32 ± 2.82	25.23 ± 2.48	0.999 §
Gender (*n* (%))						0.985
Female	133 (69%)	34 (71%)	34 (71%)	32 (67%)	33 (69%)	
Male	59 (31%)	14 (29%)	14 (29%)	16 (33%)	15 (31%)	
Alcohol Use (*n* (%))						0.706
Daily	5 (3%)	0 (0%)	2 (4%)	2 (4%)	1 (2%)	
None	32 (17%)	9 (19%)	5 (10%)	9 (19%)	9 (19%)	
Occasionally	100 (52%)	27 (56%)	25 (52%)	21 (44%)	27 (56%)	
Weekly	55 (29%)	12 (25%)	16 (33%)	16 (33%)	11 (23%)	
Smoking Status (*n* (%))						0.673
Ex-Smoker	25 (13%)	7 (15%)	8 (17%)	3 (6%)	7 (15%)	
Non-Smoker	145 (76%)	34 (71%)	36 (75%)	40 (83%)	35 (73%)	
Current Smoker	22 (11%)	7 (15%)	4 (8%)	5 (10%)	6 (12%)	
Race (*n* (%))						0.835
Black or African-American	2 (1%)	2 (4%)	0 (0%)	0 (0%)	0 (0%)	
Central American	4 (2%)	2 (4%)	1 (2%)	0 (0%)	1 (2%)	
East Asian	4 (2%)	1 (2%)	0 (0%)	1 (2%)	2 (4%)	
Eastern European White	8 (4%)	0 (0%)	3 (6%)	0 (0%)	5 (10%)	
Middle Eastern	6 (3%)	2 (4%)	1 (2%)	2 (4%)	1 (2%)	
North American Indian	2 (1%)	0 (0%)	0 (0%)	2 (4%)	0 (0%)	
South American	12 (6%)	0 (0%)	6 (12%)	3 (6%)	3 (6%)	
South Asian	5 (3%)	3 (6%)	1 (2%)	0 (0%)	1 (2%)	
South East Asian	2 (1%)	2 (4%)	0 (0%)	0 (0%)	0 (0%)	
Western European White	147 (77%)	36 (75%)	36 (75%)	40 (83%)	35 (73%)	
Ethnicity (*n* (%))						0.841
Hispanic or Latino	18 (9%)	3 (6%)	6 (12%)	4 (8%)	5 (10%)	
Not Hispanic or Latino	174 (91%)	45 (94%)	42 (88%)	44 (92%)	43 (90%)	

*n*—number of participants; BMI—body mass index; PDX—polydextrose; Max—maximum; Min—minimum; SD—standard deviation; §—between-group comparisons by ANOVA; σ—between-group comparisons by chi-square test.

**Table 2:** Average 3-day food record results previous to baseline (Day 0) and previous to end of study (Day 15) for participants in the intention-to-treat population (*n* = 192).

	12 g PDX	8 g PDX	4 g PDX	Placebo	*p*-Value Δ
	Mean ± SD (*n*) within Group *p*-Value δ	Mean ± SD (*n*) within Group *p*-Value δ	Mean ± SD (*n*) within Group *p*-Value δ	Mean ± SD (*n*) within Group *p*-Value δ	
Total Energy (kcal)					
Baseline	1707 ± 494 (48)	1712 ± 486 (48)	1822 ± 531 (48)	1766 ± 560 (48)	-
End of Study	1695 ± 520 (48)	1698 ± 529 (48)	1723 ± 448 (46)	1649 ± 500 (48)	0.709 *
Change from Day 0 to Day 15	−12 ± 435 (48) *p* = 0.686 *	−15 ± 375 (48) *p* = 0.657 *	−99 ± 400 (46) *p* = 0.132 *	−117 ± 404 (48) *p* = 0.065 *	-
Protein Mass (g)					
Baseline	75.1 ± 34.3 (48)	76.0 ± 27.8 (48)	75.5 ± 27.6 (48)	76.7 ± 23.2 (48)	-
End of Study	75.2 ± 28.9 (48)	75.0 ± 27.6 (48)	73.5 ± 27.3 (46)	70.6 ± 27.3 (48)	0.205 *
Change from Day 0 to Day 15	0.1 ± 23.3 (48) *p* = 0.585 *	−1.0 ± 18.9 (48) *p* = 0.810 *	−1.8 ± 21.8 (46) *p* = 0.580 *	−6.1 ± 24.0 (48) *p* = 0.027 *	-
Carbohydrate Mass (g)					
Baseline	207 ± 72 (48)	202 ± 69 (48)	219 ± 77 (48)	209 ± 76 (48)	-
End of Study	199 ± 80 (48)	207 ± 82 (48)	209 ± 75 (46)	204 ± 78 (48)	0.764 *
Change from Day 0 to Day 15	−8 ± 68 (48) *p* = 0.182 *	5 ± 56 (48) *p* = 0.887 *	−10 ± 48 (46) *p* = 0.177 *	−5 ± 63 (48) *p* = 0.503 *	-
Fibre Mass (g)					
Baseline	17.7 ± 9.6 (48)	18.0 ± 8.4 (48)	18.0 ± 9.1 (48)	16.8 ± 7.4 (48)	-
End of Study	16.5 ± 7.8 (48)	16.8 ± 7.4 (48)	16.4 ± 7.4 (46)	16.5 ± 7.3 (48)	0.962 *
Change from Day 0 to Day 15	−1.3 ± 7.7 (48) *p* = 0.357 *	−1.2 ± 6.6 (48) *p* = 0.198 *	−1.4 ± 6.0 (46) *p* = 0.266 *	−0.3 ± 7.1 (48) *p* = 0.779 *	-
Lipid Mass (g)					
Baseline	64.9 ± 23.1 (48)	66.2 ± 27.5 (48)	71.7 ± 29.2 (48)	67.2 ± 29.5 (48)	-
End of Study	65.2 ± 23.2 (48)	61.6 ± 22.2 (48)	65.8 ± 25.0 (46)	60.1 ± 23.5 (48)	0.567 *
Change from Day 0 to Day 15	0.3 ± 26.1 (48) *p* = 0.916 *	−4.7 ± 23.5 (48) *p* = 0.237 *	−6.2 ± 26.8 (46) *p* = 0.187 *	−7.1 ± 24.3 (48) *p* = 0.106 *	-
Water Mass (g)					
Baseline	1389 ± 800 (48)	1356 ± 760 (48)	1563 ± 846 (48)	1645 ± 809 (48)	-
End of Study	1344 ± 728 (48)	1290 ± 901 (48)	1375 ± 746 (46)	1528 ± 888 (48)	0.752*
Change from Day 0 to Day 15	−46 ± 709 (48) *p* = 0.927 *	−66 ± 516 (48) *p* = 0.067 *	−157 ± 798 (46) *p* = 0.217 *	−117 ± 848 (48) *p* = 0.110 *	-

Δ—between-group comparisons by ANCOVA, adjusting for the baseline; δ—within-group comparisons by paired Student’s *t*-test; *—logarithmic transformation required to achieve normality.

**Table 3:** Total colonic transit time results at baseline (Day 0) and end of study (Day 15) for participants in the intention-to-treat population (*n* = 192).

	All	12 g PDX	8 g PDX	4 g PDX	Placebo	*p*-Value Δ
	Mean ± SD (*n*) within Group *p*-Value δ	Mean ± SD (*n*) within Group *p*-Value δ	Mean ± SD (*n*) within Group *p*-Value δ	Mean ± SD (*n*) within Group *p*-Value δ	Mean ± SD (*n*) within Group *p*-Value δ	
Baseline	42 ± 32 (192)	39 ± 26 (48)	40 ± 31 (48)	46 ± 31 (48)	43 ± 39 (48)	0.779 ^λ^ §
End of Study	44 ± 35 (192)	38 ± 28 (48)	44 ± 35 (48)	50 ± 43 (48)	45 ± 31 (48)	0.328 λ
Change from Day 0 to Day 15	2.1 ± 24.8 (192) *p* = 0.569 λ	−1.8 ± 23.9 (48) *p* = 0.268 λ	4.2 ± 23.6 (48) *p* = 0.409 λ	4.2 ± 23.9 (48) *p* = 0.698 λ	1.7 ± 27.9 (48) *p* = 0.298 λ	0.328 λ

Δ—Between-group comparisons by ANCOVA, adjusting for baseline and age; §—between-group comparisons by ANOVA; δ—within-group comparisons by paired Student’s *t*-test; λ—square root transformation required to achieve normality.

**Table 4:** Patient assessment of constipation symptoms (PAC-SYM) and quality of life (PAC-QoL) at baseline (Day 0) and end of study (Day 15) for participants in the intention-to-treat population (*n* = 192).

	All	12 g PDX	8 g PDX	4 g PDX	Placebo	*p*-Value ^Δ^
	Mean ± SD (*n*) within Group *p*-Value δ	Mean ± SD (*n*) within Group *p*-Value δ	Mean ± SD (*n*) within Group *p*-Value δ	Mean ± SD (*n*) within Group *p*-Value δ	Mean ± SD (*n*) within Group *p*-Value δ	
PAC-SYM Total Score						
Baseline	1.07 ± 0.56 (192)	1.11 ± 0.71 (48)	1.01 ± 0.47 (48)	1.08 ± 0.54 (48)	1.08 ± 0.51 (48)	0.928 λ §
End of Study	0.90 ± 0.54 (192)	0.89 ± 0.60 (48)	0.83 ± 0.48 (48)	1.03 ± 0.57 (48)	0.86 ± 0.51 (48)	0.202 λ
Change from Day 0 to Day 15	−0.17 ± 0.50 (192) *p* < 0.001	−0.23 ± 0.58 (48) *p* = 0.004 λ	−0.18 ± 0.42 (48) *p* = 0.003 λ	−0.05 ± 0.42 (48) *p* = 0.315 λ	−0.22 ± 0.54 (48) *p* = 0.004 λ	0.202 λ
PAC-SYM Abdominal Symptoms Score						
Baseline	1.15 ± 0.69 (192)	1.18 ± 0.82 (48)	1.07 ± 0.61 (48)	1.19 ± 0.65 (48)	1.16 ± 0.69 (48)	0.926 λ §
End of Study	1.04 ± 0.71 (192)	1.05 ± 0.77 (48)	0.97 ± 0.72 (48)	1.16 ± 0.66 (48)	0.97 ± 0.68 (48)	0.406 λ
Change from Day 0 to Day 15	−0.11 ± 0.58 (192) *p* = 0.008	−0.14 ± 0.67 (48) *p* = 0.055 λ	−0.09 ± 0.51 (48) *p* = 0.014 λ	−0.03 ± 0.54 (48) *p* = 0.531 λ	−0.19 ± 0.59 (48) *p* = 0.016 λ	0.406 λ
PAC-SYM Rectal Symptoms Score						
Baseline	0.58 ± 0.65 (192)	0.69 ± 0.82 (48)	0.55 ± 0.59 (48)	0.54 ± 0.62 (48)	0.56 ± 0.53 (48)	0.866 λ §
End of Study	0.44 ± 0.54 (192)	0.43 ± 0.46 (48) a,b	0.33 ± 0.42 (48) a,b	0.61 ± 0.71 (48) a	0.37 ± 0.50 (48) b	0.034 λ
Change from Day 0 to Day 15	−0.15 ± 0.60 (192) *p* = 0.001	−0.26 ± 0.69 (48) a,b *p* = 0.035 λ	−0.22 ± 0.52 (48) a,b *p* = 0.011 λ	0.07 ± 0.57 (48) a *p* = 0.488 λ	−0.19 ± 0.58 (48) b *p* = 0.017 λ	0.034 λ
PAC-SYM Stool Symptoms Score						
Baseline	1.48 ± 0.73 (192)	1.48 ± 0.88 (48)	1.41 ± 0.59 (48)	1.52 ± 0.73 (48)	1.52 ± 0.70 (48)	0.787 λ §
End of Study	1.24 ± 0.77 (192)	1.19 ± 0.84 (48)	1.20 ± 0.76 (48)	1.34 ± 0.69 (48)	1.23 ± 0.78 (48)	0.706 λ
Change from Day 0 to Day 15	−0.24 ± 0.72 (192) *p* < 0.001	−0.28 ± 0.82 (48) *p* = 0.0012 λ	−0.22 ± 0.68 (48) *p* = 0.008 λ	−0.19 ± 0.59 (48) *p* = 0.029 λ	−0.29 ± 0.80 (48) *p* = 0.007 λ	0.706 λ
PAC-QoL Overall Score						
Baseline	1.10 ± 0.43 (192)	1.16 ± 0.48 (48)	1.00 ± 0.44 (48)	1.14 ± 0.41 (48)	1.10 ± 0.38 (48)	0.273 λ §
End of Study	1.03 ± 0.40 (192)	1.12 ± 0.46 (48)	0.95 ± 0.36 (48)	1.06 ± 0.40 (48)	1.00 ± 0.38 (48)	0.470 λ
Change from Day 0 to Day 15	−0.07 ± 0.37 (192) *p* = 0.008 λ	−0.04 ± 0.35 (48) *p* = 0.642 λ	−0.05 ± 0.44 (48) *p* = 0.546 λ	−0.08 ± 0.31 (48) *p* = 0.078 λ	−0.11 ± 0.37 (48) *p* = 0.039 λ	0.470 λ
PAC-QoL Worries and Concerns Score						
Baseline	1.06 ± 0.68 (192)	1.16 ± 0.77 (48)	0.89 ± 0.61 (48)	1.17 ± 0.74 (48)	1.01 ± 0.58 (48)	0.187 λ §
End of Study	0.88 ± 0.63 (192)	0.90 ± 0.65 (48)	0.70 ± 0.56 (48)	1.02 ± 0.68 (48)	0.88 ± 0.62 (48)	0.312 λ
Change from Day 0 to Day 15	−0.18 ± 0.56 (192) *p* < 0.001 λ	−0.26 ± 0.62 (48) *p* = 0.005 λ	−0.19 ± 0.52 (48) *p* = 0.004 λ	−0.14 ± 0.45 (48) *p* = 0.046 λ	−0.13 ± 0.64 (48) *p* = 0.087 λ	0.312λ
PAC-QoL Physical Discomfort Score						
Baseline	1.29 ± 0.73 (192)	1.41 ± 0.80 (48)	1.14 ± 0.71 (48)	1.34 ± 0.66 (48)	1.28 ± 0.73 (48)	0.358 λ §
End of Study	1.09 ± 0.79 (192)	1.18 ± 0.87 (48)	0.92 ± 0.76 (48)	1.20 ± 0.76 (48)	1.05 ± 0.73 (48)	0.372 λ
Change from Day 0 to Day 15	−0.20 ± 0.69 (192) *p* < 0.001 λ	−0.23 ± 0.73 (48) *p* = 0.008 λ	−0.22 ± 0.72 (48) *p* = 0.019 λ	−0.14 ± 0.56 (48) *p* = 0.056 λ	−0.23 ± 0.75 (48) *p* = 0.047 λ	0.372 λ
PAC-QoL Psychosocial Discomfort Score						
Baseline	0.58 ± 0.59 (192)	0.61 ± 0.58 (48)	0.47 ± 0.53 (48)	0.66 ± 0.66 (48)	0.59 ± 0.58 (48)	0.411 λ §
End of Study	0.46 ± 0.57 (192)	0.52 ± 0.67 (48)	0.32 ± 0.42 (48)	0.51 ± 0.53 (48)	0.50 ± 0.60 (48)	0.502 λ
Change from Day 0 to Day 15	−0.12 ± 0.56 (192) *p* = 0.003λ	−0.08 ± 0.57 (48) *p* = 0.060λ	−0.15 ± 0.60 (48) *p* = 0.062λ	−0.15 ± 0.49 (48) *p* = 0.115λ	−0.09 ± 0.56 (48) *p* = 0.106 λ	0.502 λ
PAC-QoL Satisfaction Score						
Baseline	1.48 ± 0.69 (192)	1.48 ± 0.74 (48)	1.50 ± 0.77 (48)	1.40 ± 0.63 (48)	1.53 ± 0.61 (48)	0.805 λ §
End of Study	1.70 ± 0.71 (192)	1.88 ± 0.75 (48) a	1.85 ± 0.73 (48) a	1.49 ± 0.72 (48) a	1.56 ± 0.58 (48) a	0.006 λ
Change from Day 0 to Day 15	0.22 ± 0.80 (192) *p* = 0.001λ	0.40 ± 1.00 (48) a *p* = 0.010λ	0.35 ± 0.78 (48) a *p* = 0.004λ	0.10 ± 0.66 (48) a *p* = 0.555λ	−0.03 ± 0.66 (48) a *p* = 0.697 λ	0.006 λ

Δ—Between-group by ANCOVA, adjusting for baseline and age; §—between-group by ANOVA; δ—within-group by paired Student’s *t*-test; λ—square root transformation to achieve normality. Superscripts (a, b) are significantly different with Dunnett’s correction.

**Table 5:** Average bowel function index questionnaire results at baseline (Day 0) and end of study (Day 15) for participants in the intention-to-treat population (*n* = 192).

	All	12 g PDX	8 g PDX	4 g PDX	Placebo	*p*-Value Δ
	Mean ± SD (*n*) within Group *p*-Value δ	Mean ± SD (*n*) within Group *p*-Value δ	Mean ± SD (*n*) within Group *p*-Value δ	Mean ± SD (*n*) within Group *p*-Value δ	Mean ± SD (*n*) within Group *p*-Value δ	
Baseline	44.9 ± 25.6 (192)	44.0 ± 28.1 (48)	40.0 ± 25.3 (48)	48.1 ± 26.6 (48)	47.6 ± 21.8 (48)	0.384 λ §
End of Study	36.7 ± 25.0 (192)	36.2 ± 26.0 (48)	34.2 ± 27.2 (48)	39.9 ± 24.0 (48)	36.4 ± 23.3 (48)	0.867 λ
Change from Day 0 to Day 15	−8.6 ± 21.7 (192) *p* < 0.001 λ	−7.9 ± 24.0 (48) *p* = 0.028 λ	−7.2 ± 21.1 (48) *p* = 0.024 λ	−8.2 ± 19.3 (48) *p* = 0.005 λ	−11.2 ± 22.6 (48) *p* = 0.001 λ	0.867 λ

Δ—Between-group comparisons by ANCOVA, adjusting for baseline and age; §—between-group comparisons by ANOVA; δ—within-group comparisons by paired Student’s *t*-test; λ—square root transformation required to achieve normality.

**Table 6:** Response to the relief of constipation questionnaire at baseline (Day 0) and end of study (Day 15) for participants in the intention-to-treat population (*n* = 192).

		All	12 g PDX	8 g PDX	4 g PDX	Placebo	*p*-Value Δ
		*n* (%) within Group *p*-Value σ	*n* (%) within Group *p*-Value σ	*n* (%) within Group *p*-Value σ	*n* (%) within Group *p*-Value σ	*n* (%) within Group *p*-Value σ	
Baseline	No Yes	95 (50%) 96 (50%)	22 (46%) 26 (54%)	20 (42%) 28 (58%)	25 (52%) 23 (48%)	28 (60%) 19 (40%)	0.328
End of Study	No Yes	66 (34%) 126 (66%) *p* = 0.002	10 (21%) a 38 (79%) *p* = 0.009	14 (29%) a 34 (71%) *p* = 0.200	24 (50%) a 24 (50%) *p* = 0.838	18 (38%) a 30 (62%) *p* = 0.031	0.020

Δ—Between-group comparisons by chi-squared test; σ—within-group comparisons by chi-squared test. Superscripts (a) are significantly different with Bonferroni correction relative to placebo.

**Table 7:** Bowel habits for baseline (run-in), week 1, and week 2 for participants in the intention-to-treat population (*n* = 192).

	All	12 g PDX	8 g PDX	4 g PDX	Placebo	*p*-Value Δ
	Mean ± SD (*n*) Within Group *p*-Value δ	Mean ± SD (*n*) Within Group *p*-Value δ	Mean ± SD (*n*) Within Group *p*-Value δ	Mean ± SD (*n*) Within Group *p*-Value δ	Mean ± SD (*n*) Within Group *p*-Value δ	
Average Weekly Number of Bowel Movements						
Run-in	8.7 ± 4.6 (192)	8.5 ± 4.1 (48)	8.8 ± 4.6 (48)	7.8 ± 4.3 (48)	9.7 ± 5.3 (48)	0.196 * §
Week 1	8.7 ± 4.7 (192)	8.8 ± 4.6 (48)	8.8 ± 4.5 (48)	7.9 ± 4.1 (48)	9.4 ± 5.4 (48)	0.918 *
Week 2	8.6 ± 4.5 (192)	9.6 ± 5.2 (48) a	8.6 ± 4.0 (48) a,b	7.4 ± 3.7 (48) a,b	8.8 ± 5.0 (48) b	0.017 *
Change from Run-in to Week 1	0.00 ± 2.87 (192) *p* = 0.971 *	0.25 ± 2.70 (48) *p* = 0.659 *	0.02 ± 2.87 (48) *p* = 0.955 *	0.08 ± 2.47 (48) *p* = 0.671 *	−0.31 ± 3.41 (48) *p* = 0.407 *	0.918 *
Change from Run-in to Week 2	−0.1 ± 3.2 (192) *p* = 0.550 *	1.1 ± 3.3 (48) a *p* = 0.059 *	−0.1 ± 3.3 (48) a,b *p* = 0.987 *	−0.4 ± 2.1 (48) a,b *p* = 0.514 *	−1.0 ± 3.7 (48) b *p* = 0.031 *	0.017 *
Average Stool Consistency						
Run-in	3.29 ± 1.05 (192)	3.42 ± 1.23 (48)	3.36 ± 1.00 (48)	3.06 ± 0.92 (48)	3.31 ± 1.03 (48)	0.359 §
Week 1	3.36 ± 1.05 (192)	3.47 ± 1.11 (48)	3.26 ± 1.07 (48)	3.40 ± 1.01 (48)	3.30 ± 1.04 (48)	0.338
Week 2	3.41 ± 1.01 (192)	3.69 ± 0.85 (48)	3.32 ± 0.94 (48)	3.30 ± 0.99 (48)	3.33 ± 1.20 (48)	0.199
Change from Run-in to Week 1	0.07 ± 0.98 (192) *p* = 0.329	0.05 ± 0.98 (48) *p* = 0.720	−0.10 ± 0.96 (48) *p* = 0.465	0.33 ± 0.96 (48) *p* = 0.019	0.01 ± 1.01 (48) *p* = 0.971	0.338
Change from Run-in to Week 2	0.12 ± 1.00 (192) *p* = 0.087	0.27 ± 1.01 (48) *p* = 0.068	−0.04 ± 0.99 (48) *p* = 0.780	0.24 ± 0.95 (48) *p* = 0.083	0.02 ± 1.04 (48) *p* = 0.889	0.199
Average Degree of Straining						
Run-in	2.13 ± 0.73 (192)	2.11 ± 0.76 (48)	2.21 ± 0.82 (48)	2.19 ± 0.68 (48)	2.02 ± 0.66 (48)	0.616 * §
Week 1	2.01 ± 0.74 (192)	2.05 ± 0.86 (48)	1.99 ± 0.71 (48)	2.04 ± 0.77 (48)	1.95 ± 0.64 (48)	0.922 *
Week 2	1.88 ± 0.71 (192)	1.83 ± 0.72 (48)	1.78 ± 0.81 (48)	2.01 ± 0.60 (48)	1.92 ± 0.68 (48)	0.065 *
Change from Run-in to Week 1	−0.13 ± 0.74 (192) *p* = 0.010*	−0.06 ± 0.80 (48) *p* = 0.492*	−0.22 ± 0.89 (48) *p* = 0.080*	−0.15 ± 0.78 (48) *p* = 0.125*	−0.07 ± 0.44 (48) *p* = 0.254*	0.922*
Change from Run-in to Week 2	−0.25 ± 0.73 (192) *p* < 0.001 *	−0.29 ± 0.65 (48) *p* = 0.002 *	−0.44 ± 0.94 (48) *p* < 0.001 *	−0.18 ± 0.63 (48) *p* = 0.072 *	−0.10 ± 0.65 (48) *p* = 0.218 *	0.065 *
Average Proportion of Complete Bowel Movements (%)						
Run-in	53 ± 33 (192)	54 ± 35 (48)	50 ± 34 (48)	49 ± 32 (48)	58 ± 31 (48)	0.520 §
Week 1	57 ± 32 (192)	61 ± 33 (48)	56 ± 33 (48)	53 ± 31 (48)	60 ± 33 (48)	0.809
Week 2	58 ± 33 (192)	64 ± 34 (48)	60 ± 35 (48)	56 ± 32 (48)	54 ± 33 (48)	0.084
Change from Run-in to Week 1	4.5 ± 28.7 (192) *p* = 0.031	6.8 ± 31.1 (48) *p* = 0.138	5.6 ± 27.3 (48) *p* = 0.163	3.6 ± 29.1 (48) *p* = 0.392	2.0 ± 27.7 (48) *p* = 0.620	0.809
Change from Run-in to Week 2	5.6 ± 29.4 (192) *p* = 0.009	9.4 ± 28.7 (48) *p* = 0.027	10.5 ± 25.8 (48) *p* = 0.007	6.8 ± 29.6 (48) *p* = 0.121	−4.4 ± 31.7 (48) *p* = 0.337	0.084

Δ—Between-group comparisons by ANCOVA, adjusting for baseline and age; §—between-group comparisons by ANOVA; δ—within-group comparisons by paired Student’s *t*-test; *—logarithmic transformation to achieve normality. Superscripts (a, b) are significantly different with Dunnett’s correction.

## Abbreviations

AEs	adverse events
BFI	Bowel Function Index
BMF	bowel movement frequency
BSS	Bristol stool scale
CSBM	Complete spontaneous bowel movements
CTT	colonic transit time
EMA	European Medicines Agency
IPAQ	International Physical Activity Questionnaire
PAC-QoL	Patient Assessment of Constipation Quality of Life
PAC-SYM	Patient Assessment of Constipation Symptoms
PDX	polydextrose
